# Top Mobile Applications in Pediatrics and Children’s Health: Assessment and Intelligent Analysis Tools for a Systematic Investigation

**DOI:** 10.21315/mjms2019.26.1.2

**Published:** 2019-02-28

**Authors:** Seyed Mohamad Hosein Mousavi Jazayeri, Amir Jamshidnezhad

**Affiliations:** 1 Nutrition and Metabolic Disease Research Center, Ahvaz Jundishapur University of Medical Sciences, Ahvaz, Iran; 2 Department of Health Information Technology, Ahvaz Jundishapur University of Medical Sciences, Ahvaz, Iran

**Keywords:** mobile health apps, clinical decision support, diagnostics, pediatrics, artificial intelligence

## Abstract

The development of intelligent software in recent years has grown rapidly. Mobile health has become a field of interest as a tool for childcare, especially as a means for parents of children with diverse diseases and a resource to promote their health conditions. Current systematic review was conducted to survey the functionalities of available applications on the mobile platform to support pediatrics intelligent diagnosis and children healthcare. Results which met the inclusion criteria (such as patient monitoring, decision support, diagnosis support) were obtained, assessed and organised into a checklist. In this study, 379 potential apps were identified using the search feature in Apple App Store and Google Play Store. After careful consideration of the selected apps, only three (Google Play Store) and one (iTunes Store), fulfilled all the general inclusion criteria and special criteria, such as intelligence tools. The results showed that Artificial Intelligence (AI) was used minimally in diagnostic apps due to a limited amount of mobile hardware and software, such as the reliable programming of intelligent algorithms.

## Introduction

Today, the use of mobile phones has become a critical component in daily use. The dependency on mobile technology and the impacts on our daily life has become a subject of research, especially the effectiveness of mobile phone use for patient care and the biological affect associated (1). The development of technology has transformed the healthcare sector and has provided access to more customised, participatory and preventative services. Mobile applications and evolving technologies offer a promising future for newer apps as a tool to assess health conditions such as Cerebral Palsy (2, 3). With the exponential rise and diversity of health apps (3), interest research in areas in promoting health, wellness and disease management has skyrocketed the past five years (4). Several accounts of intervention studies based on mobile apps have focused on specific medical issues, i.e. diabetes (5, 6) cancer (7), and weight management (8, 9).

Mobiles apps are not limited to assessing diseases and tracking health and diagnosis within the medical field. Smartphones can now be used for administrative purposes such as seamlessly communicating with patients. Some features include: tracking billing and health information, scheduling appointments, checking laboratory results and clinical decision support (CDS) (10). Apps are constantly evolving and limitations exist. Providing smartphone-based CDS can be challenging dependent upon phone type, platform, operating system, communication of the application and various device-data integrations (11).

AI or Artificial Intelligence (12), has gained interest in advancing technologies within the medical field such as Stroke Imaging (13), Medical Imaging (14), and analysing complex medical datasets (15). Stroke patients can now receive detailed and individualised information provided by AI, which determine the prognosis and therapeutic methods needed (13). AI has advanced the development of more effective and robust obesity intervention programmes (16), which compute cognitive and e-Science research models that can augment and amplify human task performance.

### Artificial Intelligence

Computer Science is advancing at ultra-fast computing speeds. AI or Artificial Intelligence continues to play a crucial role in modern society, including weather forecasting, facial recognition, fraud detection, and understanding genomics. The future role of AI in medicine and practice has yet to reveal its potential. Computers or machines, can recognised organised systems or patterns often difficult to decode using biostatistics to produce and process massive datasets, or big data, using multilayered mathematical systems (algorithms). The correction of algorithmic errors, also known as “training” consistently develops machine intelligence or AI predictive model confidence. Intelligence built in computer programmes covered by several techniques such as Artificial Neural Networks (ANN), Fuzzy Logic, Swarm Intelligence and k-Nearest Neighborhood (k-NN). The most intelligent techniques model the learning process by mathematical functions to get the inherent advantages of intelligence (17).

Within the medical industry such as radiology, pathology, and dermatology, image analysis has been successfully achieved by AI at exceeding speeds with unparalleled accuracy. Though the AI’s diagnostic confidence is never 100%, the collaboration between machines and physicians increase the reliability of system performance (18). Medical administration has been transformed as AI are able to recognise natural language processing to identify the rapidly growing scientific literature and aggregate years of electronic medical records. Thus, AI may have the ability to recommend precision therapies for complex illnesses, minimise medical errors, better subject enrollment into clinical trials and advance the field of chronic disease patients (19).

The aim of the current study was to administer an updated and rigorous systematic review of available mobile applications based on AI tools to detail the existence of evidence-based strategies, artificial intelligence analysis tools, support diagnosis, health monitoring, and support for assessing children’s health and pediatrics for the involvement of healthcare experts and scientific evaluation. The results of this study will be utilised to pinpoint the most relevant and scientific-based apps to provide evidence and orientation towards advanced research and apps development.

## Methods

The Google Play Store (https://play.google.com/store?hl=en) and Apple iOS App Store (https://itunes.apple.com/us/genre/ios/id36?mt=8) were utilised to search for apps with specific health care criteria.

According to the requirements of this study, all apps found in a systematic search within both platforms were considered independent. Search samples were found in December 2017. The apps selected were searched within health and medical fields. A search and collection was conducted by both free and paid apps. Free apps were carefully and systematically considered according to the field of study within the medical and health categories. Paid apps were assessed and information was obtained based upon the general description provided.

Criteria for this study included: patient monitoring, decision support, diagnosis support, health monitoring and artificial intelligent analysing tools for children health assessment and pediatrics.

**Figure 1:**
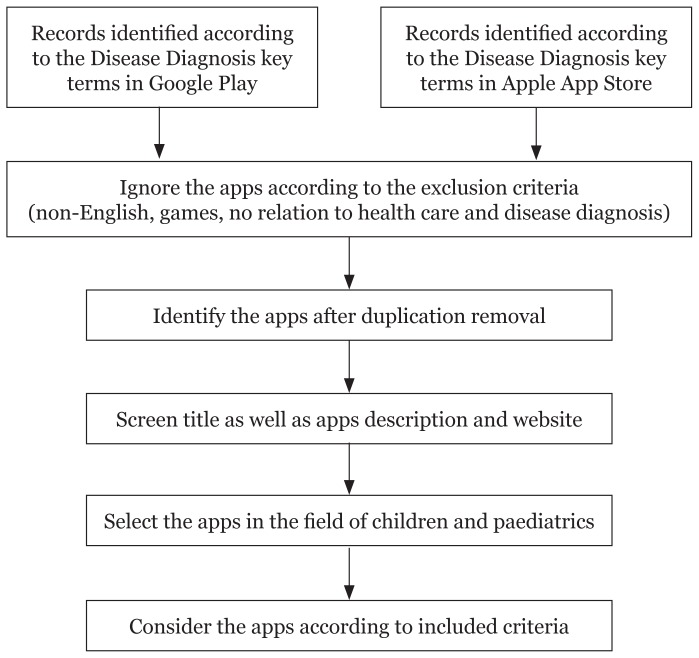
Flowchart of the selection process

Selection and assessment of apps were based upon the initial inclusion criteria such as the title and available summary descriptions. Exclusion criteria were apps unrelated to healthcare and medical services, developed as a game or were not available in English. Apps accepted into the study were considered and information was gathered to produce observations through descriptions available within the apps store and if present, on the website of the apps. The collected results were reviewed, scored, summarised and compared. Figure 1, shows the flowchart and the selection process.

### Apps Measurement Tool

Apps were analysed, measured and scored (see Table 1). The table was created based upon general criteria used in current and related studies, while more recent and newer criteria were added to provide the research in this study (2, 3, 20). The preliminary assessment was administered according to the identified and reported apps in Google Play Store and Apple iOS Store. Then, apps selected were determined for the inclusion criteria in line with general key factors. Next, apps were categorised within the scope of children and adolescent patients. Lastly, AI as a method for data analysis and diagnostic process was the ultimate target.

The population of apps users in this study included physicians and professionals in the healthcare sector, medical academic institutions and non-clinical users such as parents. Apps that had multi-target users, professional and non-clinical versions, if available, were categorised. Lastly, statistical descriptive analysis methods were used to gather data. Key points of apps were described in the comparison reports obtained from statistical analysis within the scope of healthcare.

## Results

Current systematic review was conducted to survey the functionalities of available applications on the mobile platform to support pediatrics intelligent diagnosis and children healthcare. Three hundred and seventy nine potential apps were identified using the search feature in Apple Store and Google Play Store. Eleven apps available in Google Play Store and nine apps in Apple iTunes were considered using the inclusion criteria. Exclusion of apps for both Apple iTunes and Google Play were: games, non-English language apps, and duplicates.

**Figure 2:**
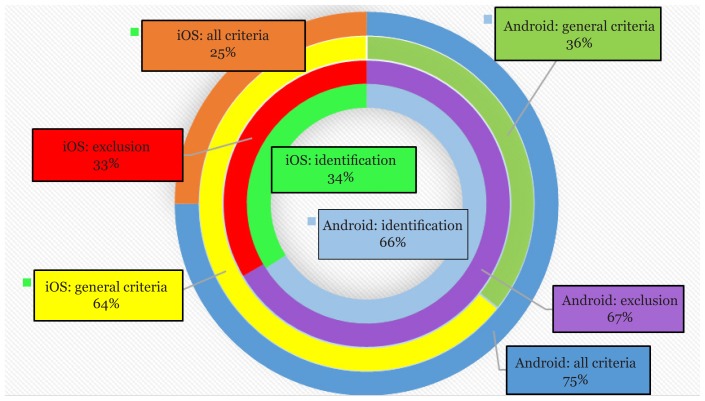
Distribution of reviewed mHealth apps in mobile markets

The majority of the apps in the search results presented one of the following services: disease text information, disease encyclopedia and dictionaries, health care education, pharmaceutical information, online patient check-up, doctors’ address information, health monitoring and patient health records. The achieved search results showed that (66%) and (34%) of the apps were available exclusively in the Android and iOS App Stores, respectively in the identification search. However, of the total apps with inclusion criteria, (36%) and (64%) of the apps met the general criteria in the Android and iOS stores. Of these, Android and iOS markets accounted for (75%) and (25%) of the apps with all functionalities. The least common criteria presented in the apps were related to the clinical decision support services. Moreover, AI methods were the least factor in special criteria. Figure 2 shows the distribution of mobile Health (mHealth) apps found in both iOS and Android markets and were reviewed in this study.

**Figure 3:**
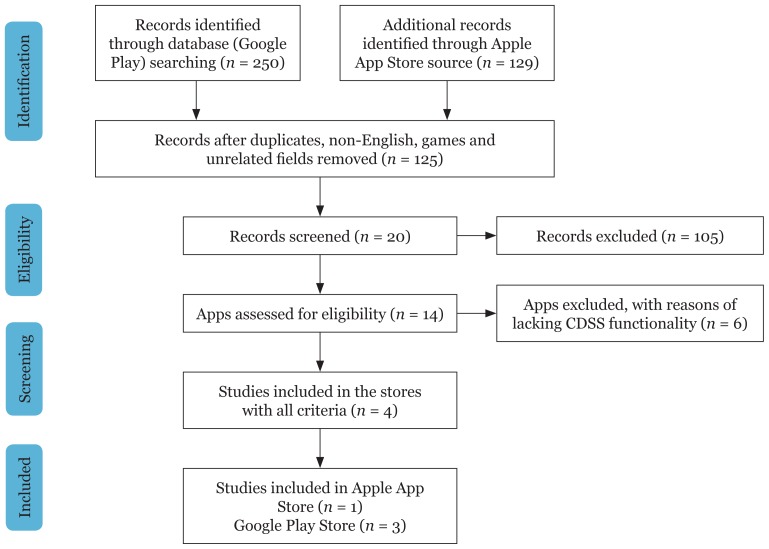
Flowchart of screening process

Figure 3 displays the screening process flowchart. Two hundred and fifty apps in Google Play Store and 129 apps in Apple Store appeared in search results. To further filter search results, non-English programmes, games and unrelated apps were eliminated in further assessments. Apps which remained were reviewed for consideration based on inclusion criteria. We found that between the apps selected, most were developed for identification or diseases, dictionaries and encyclopedia, and an online check-up and scheduling assistant. Therefore, these apps were not able to aid users as a clinical decision support system.

The results showed 14 apps were equipped with general and special criteria. After careful consideration of the selected apps, only three (Google Play Store) and one (iTunes Store), fulfilled all the general inclusion criteria and special criteria, such as intelligence tools.

Table 2 lists all apps with general inclusion and special criteria. Moreover, Table 2 reveals that apps with the iOS platform were not eligible due to the inclusion criteria, both in general and special criteria, targeted especially towards children and adolescents. It is interesting to note that Google Play Store had apps with AI methods. In conclusion, Android is a more viable platform for developers interested in exploring AI techniques in clinical decision support apps.

## Discussion

mHealth, a sector of mobile health, is an aggregation and sharing of health services and information between patients and provider via mobile and wireless devices such as phones (21). The mHealth apps is a leading resource and has transformed mobile apps services. Apps such as mHealth, have the potential to cut costs, promote patient engagement, and improve health outcomes (22). This review has provided an overview of the literature on a broad range of smartphone healthcare apps in hospital care settings.

In December 2017, this study provided a thorough review of the mHealth industry. The primary initiatives were telemedicine, disease awareness, record keeping, followed by medical education and scholarly information. A staggering majority of the apps that appeared in search results were not focused on a specific medical sector or specialty. Targeted populations for these apps were primarily non-health care professionals (HCPs). Furthermore, an overwhelming majority did not mention security measures. A detailed discussion of each apps is below.

### Platform

Based on Figure 3, apps that were AI driven and more functional, were readily available on Android than iOS platforms, though the iOS App Store is known to have more apps available in general. Both platforms had a majority of apps available in their respective mobile app stores.

### Diagnosis

We found a lack of scientific information measuring the diagnostic value of health apps present in medical literature, as stated in a recent systematic comprehensive review and preliminary meta-analysis (6). The information about the diagnostic accuracy of currently available health apps on Apple’s and Google’s App Stores is almost absent (20). As a result, apps users and healthcare professionals should be aware of the limitations when recommending specific programmes. Evidence-based and close analyses of diagnostic studies are lacking to date. The fact that the majority of apps target a diagnostic issue, it would be wise to explore an app’s scientific basis (6). Samples of available case studies were few and had very low methodological quality. For example, in reports assessing parameters of diagnostic accuracy, only one-third were available (6). A very large majority of existing health apps have not been subjected to an examination of scientific enquiry prior to launching (6).

There are over 165,000 apps related to the medical field and are available on Android and iOS mobile platforms. Of these apps, 9% address topics of screening, diagnosis and tracking of illnesses (8). Nonetheless, while many researchers predicted that mobile health apps would become transformative in the 21st century, others indicated that a strong scientifically proven background for mobile health apps continues to be sparse (9).

Generally, healthcare providers are dependable advisors on medical information and the suggestions recommended by health apps are intended to deliver health related messages to patients and their families (11).

Enabled by the Health Information Technology for Economic and Clinical Health (HITECH) Act and desire to meet meaningful use of objectives, the integration of electronic health records in primary care has not deterred the use of Clinical Decision Support (CDS) tools in practice.

Defined by Osheroff et al. (23) as “providing clinicians or patients with computer-generated clinical knowledge and patient-related information, intelligently filtered or presented at appropriate times to enhance patient care”, resources have the ability to advance clinical performance while lessening the gap in practice and knowledge and improving safety (24).

Clinical practice and guideline adherence have been demonstrated as effective through systematic reviews of CDS (25). Strong evidence has shown that CDS systems improved practitioner performance through a comprehensive evaluation of systematic reviews by Jaspers et al. (26).

Findings of each apps observed in this study are listed below:

- *Symptomate Symptom Checker*: An informational apps where users search for recommendations regarding potential clinicians, health insurance policies and pharmaceutical services. The diagnosis is organised as a list of questions about the signs and symptoms, including laboratory results. Users must be at least 18 years old with no clinical background history and may use the app to diagnosis the possible causes of their symptoms. Symptomates has over 400 conditions available to cross check the symptoms from ages 3 and up. However, the results for diagnosis from the app are unusable unless otherwise consulted by a physician.
- *US Diagnosis*: US Diagnosis, a medical diagnosis tool, utilises an extensive database with over 1,500 symptoms and causes. The primary goal of the app was to locate an observed diagnosis gathered through a survey of questions answered by users. This interactive app is available to user without prior medical knowledge and medical students. General information on symptoms from subjects ranged from ages 3 and up. US Diagnosis was created by licensed professional doctors residing in US educational medical facilities. The apps was rated 5 stars with over 50,000 downloads and demonstrates high performance in user preference.
- *OneRing—Artificial Intelligence for Parkinson’s disease*: OneRing is a tool targeted towards Parkinson’s disease and allows users to create daily reports. A ring is used to track Parkinson’s disease and can be used to assist doctors to prescribe medications. The apps can also be used without the ring tool.
- *PAIRS Medical Diagnosis*: PAIRS App was developed as a Clinical Decision Support System (CDSS). This allows for the minimisation of clinical mistakes and assist doctors. PAIRS features over 28,000 links for 486 internal diseases which include signs, symptoms and laboratory tests. The Systematized Nomenclature of Medicine is referenced as an index for medical terminology and definitions to assist in correctly interpreting patient data. PAIRS is used to diagnose patient disease for 3 years old and up.

### AI

- *Symptomate symptom*: Machine learning investigates the mechanisms by which knowledge is acquired through experience (27). Machine learning algorithms are a process or a set of rules structured in calculations or other problem solving operations, in which computing models solve complex problems, as in medical diagnosis (28). Real problems cannot accurately output as algorithms often run at a high computing cost due to complicated condition. Accuracy of diagnosis is achieved through AI techniques. In conjunction with AI, doctor knowledge was consulted in the development of the app. As a result, Symptomate is in constant improvement through its learning process, which is used to find out the main causes of the sign and symptoms with lowest risk. A rating of 4.3 stars reveals user satisfaction.
- *US Diagnosis*: US Diagnosis models doctors’ knowledge to enhance AI techniques. The app uses the AI to access an extensive database of symptoms. Information gathered from both the doctor and user are used to diagnose the symptoms. Advanced AI algorithms are used in the symptom analysis of the US Diagnosis. The AI algorithm has created an intelligent model with reasonable performance for medical diagnosis.
- *OneRing-Artificial Intelligence for Parkinson’s disease*: OneRing was the only AI app found in the Apple App Store search results. The apps utilises intelligent machine learning to synthesis and create movement patterns common in Parkinson’s disease, including dyskinesia, bradykinesia, and tremour. Moreover, automated diagnostics are conducted through an AI categorisation process. In addition, in the diagnostics process uses the mobile phone’s accelerometer through signal processing for data analysis. As a result of the OneRing App, mobile phones become powerful diagnostic support tools through combining the ring as a wearing device with integrated AI techniques.
- *PAIRS Medical Diagnosis*: NLP or Natural Language Processing was utilised to analyse user queries and convert language into medical terminology. Patient data was interpreted more accurately in clinical terms. Algorithms using NLP and Bayesian probabilistic techniques were used for diagnosis. Differential diagnosis was achieved through a Bayesian probabilistic method to group clinical factors using patients’ signs and symptoms to cluster diseases accordingly. The intelligent algorithm used in the PAIRS App revealed accurate and high performance results in the diagnosis. The probability theory within the Bayesian algorithm was used to find optimal results. Like the majority of AI processes with machine learning, the algorithm evaluates the most probably outcomes. Therefore, the Bayesian algorithm must modify the screening tool in the PAIRS App. Notwithstanding, enough information in the database and diseases must be available for the model to predict the best results.

## Conclusion

A limited amount of apps are available today which integrate and address crucial features for both paediatrics and clinical specialists such as design, development and implementation of CDS. Mobile apps offer the effective solutions to monitor the health care but are limited to diagnosis of diseases particularly for the pediatrics. Moreover, as a result of this systematic investigation, AI was used minimally in diagnostic apps due to a limited amount of mobile hardware and software, such as the reliable programming of intelligent algorithms. Lastly, apps reviewed in this study that included all criteria were not necessarily focused on pediatrics. Reframing of the research study is needed and further focused research is required in such apps with respect to their clinical implementation, end-user acceptability, evaluation by medical professionals, and security and privacy.

## Limitations

There are limitations within the study. First, the availability of the number sampled is few and does not reflect the full breadth of the market. This study is meant to provide a snapshot of apps existing within the medical industry rather than each specific specialisation. An analysis could be done by specific disease or specialty vertical to gain an understanding of the characteristics of an app. Second, included primarily medical apps rather than general health apps. Targeting medical apps provided a more focused homogenous analysis as the nature and interest of health users and sick user differ. As a result, we omitted a significant portion of the apps from the final analysis.

## Figures and Tables

**Table 1:** Apps measurement tools

General Mobile Health Care Criteria	Specific Mobile Health Care Criteria
User health record User health monitoring Disease awareness Clinical decision support Telemedicine Data analysis Diagnosis	Children and adolescents scope Intelligent algorithms for data analysis

**Table 2:** List of included apps with general inclusion and special criteria

Apps Name	Star Rating	Platform		Scope	Inclusion Criteria	

					General	AI
	
		Google Android	Apple Apps Store		General Mobile Health Care Criteria	Specific Mobile Health Care Criteria
Ada	4.7	Yes		Children and others	Yes	-
Symptomate symptom checkers	4.3	Yes		Children and others	Yes	Yes
Find TB	5	Yes		Children, adolescents and others	Yes	-
US diagnosis medical	4.2	Yes		Not mentioned specifically	Yes	Yes
PAIRS medical diagnosis	4.2	Yes		Not mentioned specifically	Yes	Yes
Uveitis doctor	No Ratings		Yes	Others	Yes	-
Oralmedx	No Ratings		Yes	Adolescents and others	Yes	-
Nephrology pocket	No Ratings		Yes	Adolescents and others	Yes	-
Diabetes clinical care	No Ratings		Yes	Adolescents and others	Yes	-
Gum disease	No Ratings		Yes	Children, adolescents and others	Yes	-
CV risk calculator	No Ratings		Yes	Children, adolescents and others	Yes	-
FH Diagnosis	No Ratings		Yes	Adolescents and others	Yes	-
Parkinson’s diseases @ point of care	No Ratings		Yes	Others	Yes	Yes
OneRing-Artificial Intelligence for Parkinson’s disease	No ratings		Yes	Children, adolescents and others	Yes	-
